# Medical genetics and genomic medicine in Chile: opportunities for improvement

**DOI:** 10.1002/mgg3.166

**Published:** 2015-07-14

**Authors:** Silvia Castillo Taucher

**Affiliations:** 1 Sección Genética, Departamento de Medicina, Hospital Clínico Universidad de ChileSantiago, Chile; 2 Sección Citogenética, Laboratorio Clínico, Clínica Alemana de SantiagoSantiago, Chile

## Geographical and Economic Data

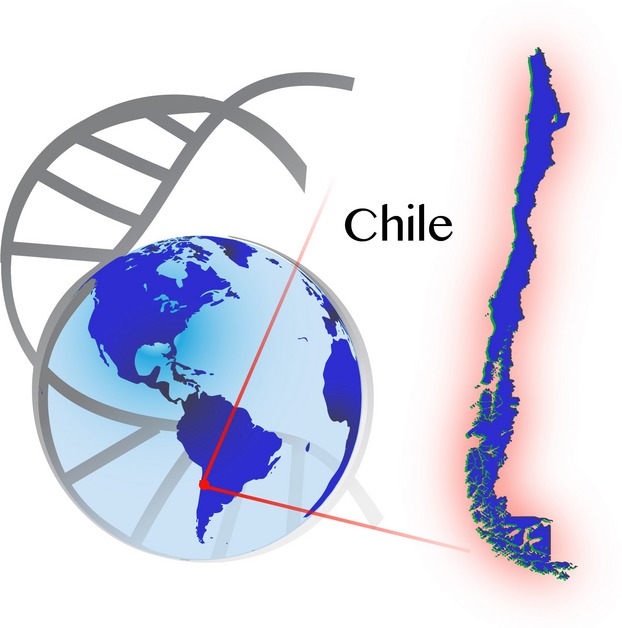



**Figure 1:**
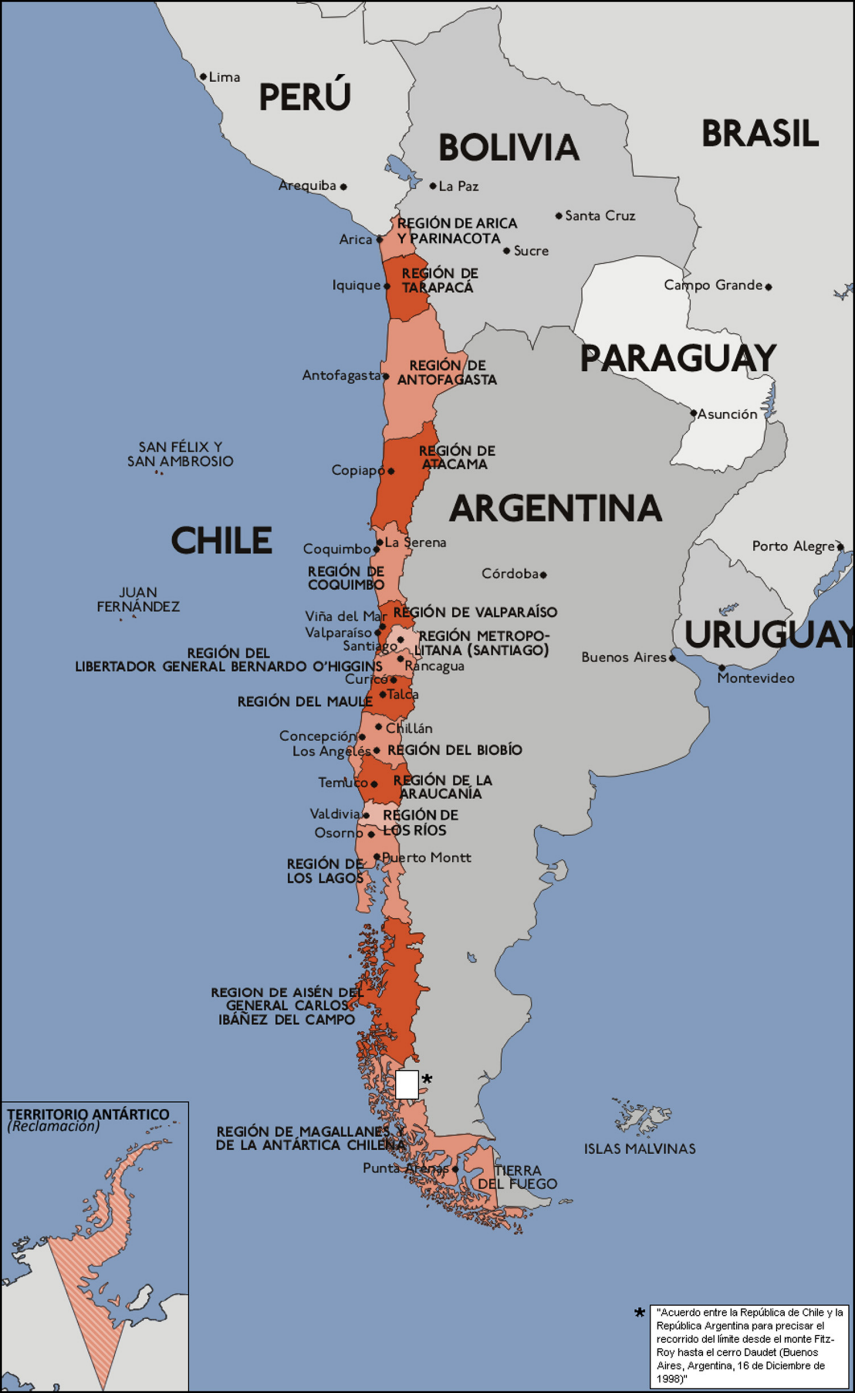
Map of Chile.

Chile, a long and narrow country in South America, is situated between the Andes mountains to the east and the Pacific Ocean to the west. It is bordered by Peru to the north, Bolivia to the northeast, Argentina to the east, and the Drake Passage in the south (Fig.1). Chilean territory includes the Pacific islands of Juan Fernández, Salas y Gómez, Desventuradas and Easter Island in Oceania. Chile claims about 1,250,000 square kilometers (480,000 sq mi) of Antarctica. Chile is the longest country in the world, with 4329 km length (2689.92 mi) (the Pacific Ocean coast stretches over 8000 km); and the narrowest, with an average width of 180 km (111.85 mi).

The northern desert of Chile has significant mineral wealth, principally copper. This relatively small central area is the cultural and political center of the country, dominating in terms of both population and agricultural resources. Southern Chile has abundant forests and grazing lands, as well as a series of volcanoes and lakes. A warren of inlets, channels, twisting peninsulas, and islands dot the southern coast. The country is divided from North to South into 15 administrative regions expanding over 292,000 mile^2^.

Spain conquered and colonized Chile in the mid-16th century, replacing Incan rule in northern and central Chile. The Spaniards were unable to conquer the independent Mapuche who inhabited south-central Chile. Since declaring its independence from Spain in 1818, Chile has developed as a relatively stable republic.

Chile leads Latin American nations in the ranking of human development, competitiveness, income per capita 15,732.31 USD (2013), globalization, the state of peace, and economic freedom. It also ranks high on the sustainability of the state, and democratic development at a regional level. Chile is a founding member of the United Nations, the Union of South American Nations and the Community of Latin American and Caribbean States (http://www.breakingnews.com/topic/chile/).

## Demographic Profile

Chile is undergoing a demographic transition and is becoming an aging society. Fertility is below replacement level with an average of 1.84 children born per woman. Mortality rates are low. Life expectancy is on par with developed countries: 78.44 years (75.42 and 81.59 years for males and females, respectively [2014 est.]). However, with its dependency ratio reaching its lowest point, Chile could benefit from a favorable age structure. It will have to maintain its large population of able bodied workers as it prepares to meet the needs of its growing proportion of older people, especially as women – the traditional caregivers – are increasingly joining the workforce. In the last two decades, Chile has made great strides in reducing its poverty rate, which is now lower than most countries in Latin America. Nevertheless, its severe income inequality ranks as the worst among members of the Organization for Economic Cooperation and Development. Unequal access to quality education continues the unequal distribution of income.

Although historically a country of emigration, Chile has gradually become more attractive to immigrants following the transition to democracy in 1990 and improved economic stability relative to other regional destinations that have simultaneously experienced worsening economic conditions and policies. The small but growing foreign population of Chile is primarily due to transplants from other Latin American countries, especially Peru. Chileans have high rates of literacy with 98.6% of the population age 15 and older being able to read and write. The official language is Spanish, which is spoken by 99.5% of residents. Approximately 10% speak English. The country is predominantly Roman Catholic (approximately 67%), but evangelical or protestant account for about 16% of the population. One percent being Jehovah’s Witnesses.

Chile’s population is 17,363,894 individuals with a median age of 33.3 years (32.2 and 34.6 years for males and females, respectively). The vast majority, approximately 89%, are white and nonindigenous. Of indigenous groups, the Mapuche are the most prevalent, accounting for approximately 9% of all individuals. The majority (89.2%) of the population lives in an urban environment. The three major cities are the capital Santiago (6,034,000), Valparaiso (883,000), and Concepcion (770,000). Dependency ratio, measure showing the number of dependents (aged 0–14 and over the age of 65) to the total population (aged 15–64), total dependency ratio: 45.1%, youth dependency ratio: 30.2%, elderly dependency ratio: 14.9%, potential support ratio: 6.7 (2014 est.). The potential support ratio (PSR) is the number of people aged 15–64 per one older person aged 65 or older. This ratio describes the burden placed on the working population (unemployment and children are not considered in this measure) by the nonworking elderly population (http://www.indexmundi.com/chile/demographics_profile.html) (INE Chile (2012); Resultados Preliminares Censo de Población y Vivienda 2012. Retrieved from: http://www.censo.cl/).

Health expenditures: 8.2% of the gross domestic product (GDP) (WHO, 2012), physicians density: 1.03 physicians/1000 population (2009), hospital bed density: two beds/1000 population (2010), maternal mortality: maternal mortality rate: 25 deaths/100,000 live births (2010). A healthcare reform in 2005 established an “explicit guarantee in health care” system (GES, “Garantías Explícitas en Salud”) with a “Universal Access Plan with Explicit Guarantees” of service (AUGE Plan) for all health insurance plans. It includes guaranteed health services for 80 diseases relevant to the Chilean population (last actualization September 2014). Among them are congenital anomalies (congenital heart disease, cleft lip and/or palate, and neural tube defect), a number of adult cancers (breast, gastric, leukemia, testicular, cervical, prostate, brain) and all infantile cancer diagnosed <15 years of age. Two monogenic diseases (hemophilia and cystic fibrosis) are also covered (http://web.minsal.cl/portal/url/item/e03c08fac00143dee0400101650176c1.pdf) (Ministry of Health, Chile (2012). Acceso Universal Garantías Explícitas (AUGE). Retrieved June 28, 2012, from http://www.redsalud.gov.cl/portal/url/page/minsalcl/g_gesauge/presentacion.html).

## Newborn Screening

In 1992, the Ministry of Health of Chile launched a national program of neonatal screening for phenylketonuria (PKU) and congenital hypothyroidism (CH), and in 1998, it was extended to the entire country. From 1992 to 2008 they analyzed a total of 2,478,123 newborns (NB), obtaining a coverage currently at 98.7% of the NB. During this period, the incidence of PKU, resulted in 1: 18,916 NB, with a mean age of diagnosis of 18 ± 10.2 days and the incidence of hyperphenylalaninemia, 1: 10,198 NB. Regarding CH the incidence is 1: 3,163 NB, with average age at diagnosis of 12.5 ± 6.9 days and the incidence of hyperthyrotrophinemia is 1: 11.634 NB (Cornejo et al. 2010). In July 2005, Law No. 19966 of the program of explicit guarantees in health, in the general system of health guarantees was approved, where the mandatory newborn screening for PKU and CH was established throughout the country (Ministry of Health 2005; www.supersalud.cl/normativa/571/articles-3174_recurso_1.pdf).

Since 1990 Extended Neonatal Screening Programs which allow diagnosis simultaneously for around 50 metabolic diseases started in USA, Australia and Germany; they have determined an overall incidence of metabolic diseases of 1:3000 live births (Kilakkathi 2012). Newborn Screening In America: Problems AND Policies. Council for Responsible Genetics www.councilforresponsiblegenetics.org). With an Extended Program, Chile could increase the actual rate of detection of 1 in 21.000 newborns, meaning around new 450 cases a year, with better chances of early treatment and decrease in mortality and morbidity from these causes.

The prevalence of genetic disorders is difficult to establish with certainty because there is currently no national registry of birth defects or official records for genetic conditions.

Data of birth defects are available since 2001 from the Maule region (south-central region of the country) and also since 1960 from some hospitals participating in the Collaborative Study of Congenital Malformations in Latin America (ECLAMC). These data suggest that the global rate of major congenital anomalies in live births is 3.1% (Castilla and Orioli 2004).

The most common congenital anomalies per 10,000 births are: congenital heart disease (57.4), talipes (20.7), polydactyly (18.6), and cleft lip and/or cleft palate (18.1). Down syndrome has an incidence of 24.7 per 10,000 (Nazer and Cifuentes 2011). Since 2000 wheat flour has been fortified with folic acid, and this is reported to have led to a decrease of 50% in the rate of neural tube defects, from 17.1 to 8.6 per 10,000 births (Cortés et al. 2012).

## National Perinatal Information System

A pilot registry of perinatal deaths (SNIP: Servicio Nacional de Información Perinatal) started in 2012 with the aims of:

1. Obtaining statistics of congenital anomalies in newborns
2. Performing surveillance of environmental causes potentially responsible for congenital anomalies
3. Delivering early alerts of new exposition to teratogens
4. Obtaining indicators of adverse pregnancy outcomes
5. Providing assessment in health planning

Thirty-five percent of neonatal deaths occur due to congenital anomalies, the most frequent causes are heart malformations, pulmonary problems, and chromosomal etiologies (trisomies 18 and 13) together with nervous system malformations (http://www.deis.cl/sistema-nacional-de-informacion-perinatal-snip/).

## National Perinatal Information System: Objectives

1. Implementing digital forms for birth and death certificates and confidentially linking information regarding birth defects
2. Gather maternal fetal information that allows knowing, monitoring, and doing follow-up of cases
3. Establishing the National Registry of Congenital Anomalies and creating the National Network of Congenital Anomalies Surveillance in Chile, REVINACH

## Genetic Counseling

In Chile, as in most Latin American countries, there are no formal postgraduate programs in genetic counseling, and no recognition of genetic counseling as an independent clinical discipline. Consequently, genetic counseling services are primarily provided by the trained clinical geneticists available and other physicians involved in the care of patients with genetic disorders. The Center for Human Genetics, Facultad de Medicina Clínica Alemana-Universidad del Desarrollo in Santiago, is the only center in the country that has a U.S.-trained, ABGC-certified genetic counselor, working since 2004 as part of the clinical, research, and educational team. She has been providing genetic counseling services for pediatrics and obstetrics and hereditary cancer risk assessment, as well as to patients and families from support organizations such as the Center of Movement Disorders for Huntington’s disease patients (CETRAM) and Dystrophic Epidermolysis Bullosa (Debra Chile). Additionally she is developing a certificate course in genetic counseling for health providers treating different areas of medicine (Margarit et al. 2013).

## Clinical Genetics

Clinical Genetics became recognized as a medical specialty in 1997. There is one clinical genetics fellowship program for physicians at Facultad de Medicina, Universidad de Chile. It has a duration of 3 years and a training program with four modules, scientific method, biological and molecular basis of genetics, laboratory genetics, and clinical genetics. Students rotate through all genetic services in Chile and are allowed to visit sites abroad. The rate of new graduates from the Chilean genetics fellowship program is 0.7 per year.

Multiple residents from other specialties like Obstetrics and Gynecology, Maternal Fetal Medicine, Endocrinology, Hemato-oncology, Neurology perform clinical genetic rotations of variable durations at University centers (Castillo Taucher 2004). The most common indications for genetic counseling are diagnosis or positive family history of chromosomal alterations, skeletal dysplasias, and recurrent miscarriages.

Chile, with its 17,819,054 inhabitants, should have an ideal number of clinical geneticists of 170 (according to WHO, one geneticist per 100,000 persons), but the real number is 30, basically one geneticist for 500,000 individuals. Twenty-two of 28 (79.1%) genetics providers reside in the capital city of Santiago, and six geneticists reside and work in the rest of the country. Clinical Genetics is considered a primary specialty in Chile, even so 12 of 28 practitioners (42.9%) are also pediatricians. The vast majority of clinical geneticists work in the public and private healthcare systems, and most see up to 70 patients per month. The key practice areas are pediatric dysmorphology and prenatal diagnosis. Many also do teaching and work in research and clinical laboratories.

## Laboratory Genetics

There are about 15 clinical cytogenetic laboratories (general regulations exist for quality and competence of all clinical laboratories) mainly offering karyotype and FISH analysis, and some molecular genetics services for a limited number of genetic diseases. Recently, chromosome microarrays (array CGH) analysis became available for clinical diagnosis (Instituto Nacional de Normalización, http://www.dipres.gob.cl/595/articles-122897_doc_pdf.pdf).

The majority of diagnoses for monogenic conditions are based on clinical evaluation. Confirmation by molecular genetic testing for certain genetic conditions can be performed by local clinical laboratories on case by case basis, and on occasion samples are sent outside of the country for genetic testing. Most genetic testing is not covered by health insurances.

Some genetic syndromes with possibilities of being studied in Chile are: fragile X syndrome, myotonic dystrophy, lactose intolerance, pharmacogenetics of oral warfarin anticoagulants, Huntington disease, cystic fibrosis, sensorineural hearing loss, alpha 1 antitrypsin deficiency, gen MTHFR, some mitochondrial diseases, achondroplasia and hypochondroplasia, thanatophoric dysplasia type I and II, spinal muscular atrophy, Prader-Willi syndrome, Angelman syndrome, Duchenne muscular dystrophy, Becker muscular dystrophy, paternity testing, multiple endocrine neoplasia RET gene, SHOX gene for short stature, hemochromatosis, hemophilia A, congenital adrenal hyperplasia related to 21-hydroxylase, Charcot-Marie-Tooth syndrome, Gilbert syndrome, Muenke craniosynostosis, Noonan syndrome, Beckwith-Wiedemann syndrome, Silver Russell syndrome, among others.

## Rare Diseases

Since January 2015 a draft legislation called Ricarte Soto is at the congress intending to take care of financial protection for expensive drugs. He was a journalist with lung cancer who fought for universal rights in these items.

Drugs, devices, and special and costly food products will be evaluated technically and scientifically, considering, medical, economic, social, and security aspects of treatments that are not covered by social security systems in health today. The recommendation for inclusion of a new treatment system will be made through a committee, with the participation of representatives of patients organizations. Every three years, a new list will be delivered, adding treatments covered by the Fund. Funding comes from direct fiscal contribution and will progressively increase.

The law provides for several instances of social participation and transparency and is of universal access (http://web.minsal.cl/leyricarte).

A center for integrated management of patients with rare diseases, (CEMINER: centro de manejo integral de pacientes con enfermedades raras), contact: ceminer@hcuch.cl, was created in 2005 for assistance in the clinical diagnosis of patients by qualified and specialized physicians safeguarding confidentiality and with an informed consent, advice in the recommendation of diagnostic tests, help in the interpretation of results of cytogenetic and molecular genetic studies, genetic counseling for patients and their families, integration of genetic knowledge, and information about support groups. We try to answer in a short time referring to a medical center or specialist nearest to the site of consultation, in Chile and sometimes abroad, to facilitate and access information relevant to the case, help to organize a plan of action and remain as a future contact.

This service complements our previous teledysmorphology site for clinical consultation of genetic syndromes allowing access to genetic services through the web from all sites of the country.

## Research

Chile is the country with the least investment in research and development of the OECD, that is, 0.4% of GDP. Even so, Chile appears as the 11th country with the highest growth in investment in research and development between 2007 and 2012 with 36% increase between the two dates. Fifty-six percent is spent in the metropolitan area.

The National Fund for Scientific and Technological Development, FONDECYT; finances numerous research projects related more or less with genetic themes, diseases of development, oncology, and specific syndromes (http://www.t13.cl/noticia/actualidad/nacional/chile-es-el-pais-con-menor-inversion-en-investigacion-y-desarrollo-de-la-ocde).

## Conclusion

Although Chile is not significantly creating new knowledge in genetics and genomics, we have good health standards and reliable services to collaborate on global projects that struggle to benefit patients and improve their quality of life.

## References

[b1] Castilla EE, Orioli IM (2004). ECLAMC: the Latin-American collaborative study of congenital malformations. Community Genet.

[b2] Castillo Taucher S (2004). Genetic services in Chile. Community Genet.

[b3] Cornejo V, Raimann E, Cabello JF, Valiente A, Becerra C, Opazo M (2010). Past, present and future of newborn screening in Chile. J. Inherit. Metab. Dis.

[b4] Cortés F, Mellado C, Pardo RA, Villarroel LA, Hertrampf E (2012). Wheat flour fortification with folic acid: changes in neural tube defects rates in Chile. Am. J. Med. Genet. A.

[b533] Kilakkathi V (2012). http://www.councilforresponsiblegenetics.org/pageDocuments/WNMAKEPP1P.pdf.

[b5] Margarit SB, Alvarado M, Alvarez K, Lay-Son G (2013). Medical genetics and genetic counseling in Chile. J. Genet. Couns.

[b6] Nazer J, Cifuentes L (2011). Malformaciones congénitas en Chile y Latino América: Una visión epidemiológica del ECLAMC del período 1995–2008. Revista Médica de Chile.

[b543] WHO (2012). http://apps.who.int/ghodata/.

